# Predicting fungal secondary metabolite activity from biosynthetic gene cluster data using machine learning

**DOI:** 10.1128/spectrum.03400-23

**Published:** 2024-01-09

**Authors:** Olivia Riedling, Allison S. Walker, Antonis Rokas

**Affiliations:** 1 Department of Biological Sciences, Vanderbilt University, Nashville, Tennessee, USA; 2 Evolutionary Studies Initiative, Vanderbilt University, Nashville, Tennessee, USA; 3 Department of Chemistry, Vanderbilt University, Nashville, Tennessee, USA; University of Wisconsin-Madison School of Medicine and Public Health, Madison, Wisconsin, USA

**Keywords:** fungi, specialized metabolism, secondary metabolism, artificial intelligence, bioactivity, drug, antibiotic, antibacterial, antifungal, cytotoxic, antitumor

## Abstract

Fungal secondary metabolites (SMs) contribute to the diversity of fungal ecological communities, niches, and lifestyles. Many fungal SMs have one or more medically and industrially important activities (e.g., antifungal, antibacterial, and antitumor). The genes necessary for fungal SM biosynthesis are typically located right next to each other in the genome and are known as biosynthetic gene clusters (BGCs). However, whether fungal SM bioactivity can be predicted from specific attributes of genes in BGCs remains an open question. We adapted machine learning models that predicted SM bioactivity from bacterial BGC data with accuracies as high as 80% to fungal BGC data. We trained our models to predict the antibacterial, antifungal, and cytotoxic/antitumor bioactivity of fungal SMs on two data sets: (i) fungal BGCs (data set comprised of 314 BGCs) and (ii) fungal (314 BGCs) and bacterial BGCs (1,003 BGCs). We found that models trained on fungal BGCs had balanced accuracies between 51% and 68%, whereas training on bacterial and fungal BGCs had balanced accuracies between 56% and 68%. The low prediction accuracy of fungal SM bioactivities likely stems from the small size of the data set; this lack of data, coupled with our finding that including bacterial BGC data in the training data did not substantially change accuracies currently limits the application of machine learning approaches to fungal SM studies. With >15,000 characterized fungal SMs, millions of putative BGCs in fungal genomes, and increased demand for novel drugs, efforts that systematically link fungal SM bioactivity to BGCs are urgently needed.

**IMPORTANCE:**

Fungi are key sources of natural products and iconic drugs, including penicillin and statins. DNA sequencing has revealed that there are likely millions of biosynthetic pathways in fungal genomes, but the chemical structures and bioactivities of >99% of natural products produced by these pathways remain unknown. We used artificial intelligence to predict the bioactivities of diverse fungal biosynthetic pathways. We found that the accuracies of our predictions were generally low, between 51% and 68%, likely because the natural products and bioactivities of only very few fungal pathways are known. With >15,000 characterized fungal natural products, millions of putative biosynthetic pathways present in fungal genomes, and increased demand for novel drugs, our study suggests that there is an urgent need for efforts that systematically identify fungal biosynthetic pathways, their natural products, and their bioactivities.

## INTRODUCTION

Fungi have captivated the scientific community for centuries due to their diverse ecological roles and remarkable ability to produce an array of bioactive secondary metabolites (SMs). SMs are small biologically active compounds that aid in adapting to different environments but are not required for normal function or survival (1). Many clades across the fungal kingdom produce SMs, including many that belong to the *Pezizomycotina* subphylum of filamentous fungi. These SMs have diverse bioactivities, including antifungals (2), UV protectants (3), antibacterials (4–6), iron sequesterers (7), antifeedants (8), immunosuppressants (9), and toxins (10). In addition to their ecological importance, fungal SMs are also of considerable interest to medicine, industry, and the bioeconomy and are used in a wide range of applications. It has been suggested that fungal SM chemical properties are more “drug-like” compared to their bacterial counterparts by US Food and Drug Administration guidelines and oral bioavailability prognostic rules (11, 12). Many SMs such as penicillin (13), an antibiotic, and lovastatin (14), a cholesterol-lowering antifungal, are iconic drugs. Other SMs have been utilized in the food science, agricultural, and cosmetic industries for bioactivities such as antibiotics, pigments, and antifeedants.

The genes involved in fungal SM production are typically located right next to each other in the genome and are known as BGCs (1, 15). It is estimated that filamentous fungal species may have up to 30–70 BGCs per genome (16), sometimes even more; these BGCs contain many different types of genes, such as core or backbone genes, whose protein products synthesize the backbone of the SM, backbone modifying genes, transport genes, transcriptional regulatory genes, and self-resistance genes whose protein products confer protection against the produced SMs (1). There are a variety of core genes, such as polyketide synthases (PKS), terpene synthases, and non-ribosomal peptide synthetases (NRPS) (16). The genes within these BGCs often vary considerably in terms of gene presence, absence, and orientation within and between species (15, 17) contributing to fungal ecological and chemical diversity (1).

Identification of novel fungal SMs is a labor-intensive process consisting of isolation, purification, and structural elucidation of novel compounds (18). Novel fungal SMs are typically subjected to bioassays for the detection of specific biological activities (e.g., antifungal or antibacterial) (19), but the lack of systematic efforts in cataloging these bioactivity data limits their potential utility in large-scale analyses. More recently, the discovery of novel SMs in diverse organisms, including bacteria, plants, and fungi, has been accelerated by advances in genomic sequencing, genetic engineering, and bioinformatics. Advances in omics technologies have enabled the discovery of novel BGC-SM pairs through genomic manipulations and the utilization of heterologous expression to isolate SMs from unculturable species (20–22) and activation or increase in SM production through promoter engineering (23).

The increasing number of characterized BGC-SM pairs from diverse organisms has led to the creation of large repositories such as the Minimum Information about a Biosynthetic Gene Cluster (MIBiG) database (24), which houses standardized annotations and metadata on BGC-SM pairs, increasing the efficiency of natural product discovery and facilitating additional analyses. In parallel, novel computational approaches, such as machine learning, are being employed to predict BGCs (25), like ClusterFinder (26); predict both BGCs and SM bioactivities, like DeepBGC (27); and predict biological activity from natural product chemistry (28, 29). For example, Walker and Clardy recently examined whether the bioactivity of natural products could be predicted from genetic data in bacteria (30).

One appeal of using machine learning is that it enables the potential identification of specific genetic or chemical features associated with different bioactivity types. Since the protein products of genes in BGCs are involved in the biosynthesis of the SM structure and structure generally determines function, we expect that genes in BGCs may give insight and/or be predictive of SM bioactivity. Machine learning models can be generally divided into two main categories, supervised and unsupervised. Supervised models rely on labeled training data as input [e.g., random forest (RF), logistic regression (LR)], whereas unsupervised ones use unlabeled raw data (e.g., neural network, hidden Markov model) to enable predictions/classifications given sufficient data. Machine learning models have been successfully used to predict both genomic features (31) and phenotypic traits (32–34) and can be used with any type of biological, including genomic, data (35).

**Fig 1:**
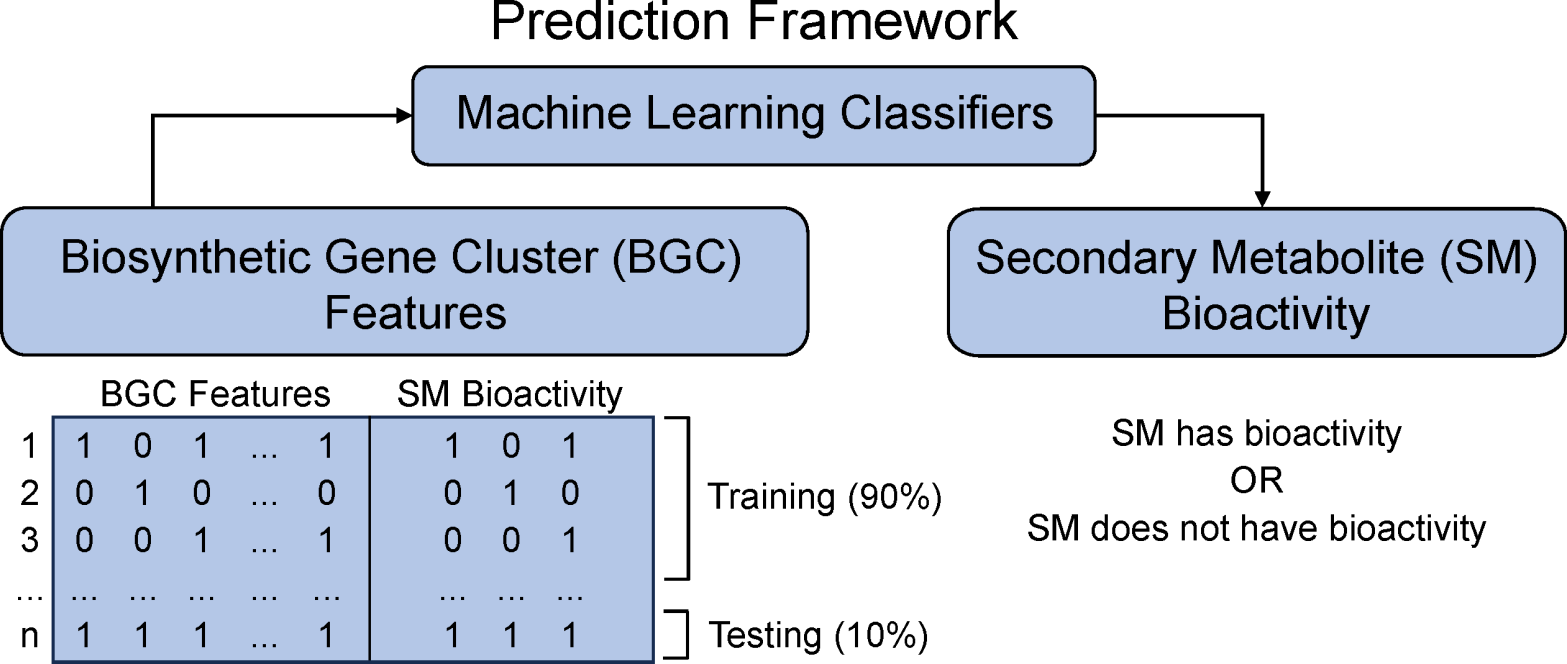
Machine learning workflow to predict fungal secondary metabolite bioactivity. We trained three classifiers (support vector machine, logistic regression, and random forest) on 90% of the biosynthetic gene cluster data and tested the classifier performance on the remaining 10% of the data using a 10-fold cross-validation approach.

In this work, we adapted machine learning models by Walker and Clardy that predict bacterial SM bioactivity from bacterial BGC data with accuracies as high as 80% (30) to test whether we could predict fungal SM bioactivity (Fig. 1). We trained the models to predict the antibacterial, antifungal, and cytotoxic/antitumor SM activity from BGC data using two training data sets: (i) fungal BGC data and (ii) fungal and bacterial BGC data. The models trained on the fungal data set (comprised of 314 BGCs) had balanced accuracies between 51% and 68%, and those trained on the fungal and bacterial data (comprised of 314 fungal and 1,003 bacterial BGCs) were between 56% and 68%. The lower balanced accuracies of our models compared to the models by Walker and Clardy (30) are likely due to the smaller size of the fungal data and the lack of informativeness of the bacterial data (for predicting fungal SM bioactivity). We conclude that the small number of fungal BGC-SM pairs with known bioactivities currently limits the use of artificial intelligence approaches for predicting SM bioactivity. Efforts that systematically catalog fungal SM bioactivity (including lack of bioactivity) and identify new BGC-SM pairs are needed.

## MATERIALS AND METHODS

### Obtaining fungal and bacterial BGC-SM pairs with known bioactivities

The fungal BGC GenBank files of 392 fungal BGCs were downloaded from the MIBiG database, version 3.0 (24). We performed a literature search of bioactivity and growth assays to identify validated bioactivities of the SM products from all 392 fungal BGCs in MIBiG. Data on the 1,152 bacterial BGCs and their SM product bioactivities were retrieved from the study by Walker and Clardy (30). SM bioactivities were categorized into “antibacterial,” “antifungal,” “cytotoxic,” “antitumor,” and “unknown” for when data were not available. We also identified SM bioactivities that were not included in our predictions (e.g., antifeedant) because of their small numbers. Bioactivity classifications were converted into binary matrices, such that “1” indicated the presence of activity and “0” indicated the absence of activity or lack of documentation. These data are available in Tables S1 and S2 (https://doi.org/10.6084/m9.figshare.24129012).

### Feature selection and construction of training data sets

Our training data included the BGC number (corresponding to the accession number of the BGC in the MIBiG database), product name, the SM product bioactivity, core gene present in the BGC that biosynthesizes the backbone of the SM product, and the species and genus classification. BGCs whose bioactivities are unknown were not included in model training. Two training data sets were used: the first contained fungal BGCs and the bioactivities of their corresponding SMs (392 BGCs in total; the SMs of 123 BGCs had antibacterial bioactivity, of which 115 had antifungal bioactivity, 96 had cytotoxic bioactivity, and 123 had antitumor bioactivity), and the second contained both fungal (392 BGCs) and bacterial BGCs (1,544 BGCs in total; the SMs of 627 BGCs had antibacterial bioactivity, of which 312 had antifungal bioactivity, 328 had cytotoxic bioactivity, and 260 had antitumor bioactivity) and the bioactivities of their SMs. From the 392 BGCs in the fungal data set, 78 were removed because their bioactivities were unknown, resulting in 314 BGCs used in training the models; similarly, from the 1,544 BGCs in the fungal and bacterial data set, 227 were removed because their bioactivities were unknown, leaving 1,317 BGCs (314 fungal and 1,003 bacterial) used in training the models.

To obtain the features for model training, each GenBank file for the BGCs was run through anti-SMASH, version 5 (36). For each BGC, we extracted from the GenBank output files generated by anti-SMASH: (i) Pfam protein family domains represented in the BGC, (ii) the core gene(s) (PKS, NRPS, etc.), (iii) cluster-defining CDS features [gene features that anti-SMASH uses to define the BGC class or classes (e.g., NRPS, PKS, etc.)], and (iv) annotations of secondary metabolite clusters of orthologous groups of proteins (smCOGs, annotations for accessory genes in BGCs based on sequence similarity to genes in other characterized BGCs). Extractions were performed using Python scripts modified from Walker and Clardy (30) (https://doi.org/10.6084/m9.figshare.24129012). To identify genes in the BGCs similar to antibiotic resistance genes, which have the potential to be predictive of antibacterial and antifungal bioactivity, the GenBank files generated by anti-SMASH, one for each BGC, were converted into fasta files and subsequently run through Resistance Gene Identifier (RGI), (version 5) (37). The RGI annotations were extracted similarly to the anti-SMASH output with a Python script, retaining only the genes that occurred five or more times in the data set (https://doi.org/10.6084/m9.figshare.24129012). All the features collected were converted into binary matrices and used in model training. These data are available in Tables S6 and S7.

### Machine learning models

To gain insights into fungal SM bioactivity, we trained machine learning models to predict three types of bioactivity: antibacterial, antifungal, and cytotoxic/antitumor (Fig. 1). Following Walker and Clardy (30), we used three algorithms to make the three binary classifications: (i) support vector clustering (SVC) module for SVM, (ii) stochastic gradient decent classifier (SGDClassifier) module for the LR, and (iii) RF with extra randomized decision trees. All three classifiers were used independently to predict each type of bioactivity since an SM can have multiple bioactivity types. Each classifier was imported from the scikit-learn Python library (38), and the parameters were determined by completing a GridSearch from scikit-learn.

The parameter values used in the GridSearch for the SVM models were *c*-values of 100, 10, 1, 0.5, 0.1, and 0.01 and gamma values of 0.01, 0.1, 1, and 10. The *c*-value is the regularization parameter that determines the hyperplane (e.g., a high *c*-value will choose a smaller-margin hyperplane focused on classifying training data points correctly and can lead to overfitting, and a small *c*-value will choose a larger-margin hyperplane to find a generalized smooth boundary but can lead to underfitting). The gamma values determine the similarity radius; a small gamma value leads to a more general model, whereas a larger gamma leads to a model more specific to the training data.

The parameter values used in the GridSearch for the LR models were maximum iterations of 100; log loss and elasticnet penalties; alpha values of 0.5, 0.3, 0.2, 0.1, 0.01, 0.001, 0.0001, 0.00001, and 0.000001; and l1 ratios of 0.5, 0.2, 0.05, 0.1, 0.01, 0.001, and 0.0001. Additionally, the tolerance was set to “none.” The alpha value is a penalty score for large correlation coefficients, which prevents the models from overfitting (e.g., higher alpha values favor simpler models with lower correlation coefficients). The l1 ratios control the balance between lasso (some coefficients are exactly zero and few features are important) and ridge (keeps the coefficients from being too large and balances the coefficients of all features) regularization, such that underfitting and overfitting are prevented. Lastly, the tolerance parameter determines the point where the model stops training based on the differences in model performance from one iteration to the next.

The parameter values for the RF models were maximum features on auto; Gini criterion; bootstraps true; maximum depth of 10, 20, 50, 100, 1,000, and none; and the number of estimators were 1, 5, 10, 15, 25, 50, and 100. The Gini criterion measures the degree of node impurity (the amount of variance in a given feature for BGCs that either have or do not have this feature) and is used to split nodes in each decision tree to reduce impurity. Maximum depth controls how deep the decision tree can grow to prevent overfitting, and the number of estimators is the number of decision trees in the forest. The best parameter values for each classifier were chosen based on the highest average accuracy.

To evaluate the models, we used a 10-fold cross-validation (10 trials, each of which holds back a random 10% of the training data for testing) to calculate the balanced accuracy metric from the scikit-learn Python library. We used balanced accuracy to address the imbalance of the bioactivity types in the training data since the bioactivities are rare and therefore have a larger portion of the 0’s compared to 1’s [e.g., there are 116 BGCs with antifungal bioactivity (1’s) compared to 198 without (0’s) in our fungal BGC training data]. Balanced accuracy takes the mean of the true positive rate (TNR) and the true negative rate to provide a more accurate representation of model performance on imbalanced data sets. The balanced accuracy of each classifier was compared to classifiers trained on randomized features that represent the inability to distinguish between classes (0 or 1). A balanced accuracy score of 50% means that the classifier predicts the correct classification as well as randomly guessing.

Each classifier for each classification was assessed using a one-way ANOVA to determine if there was a significant difference between using the actual training data vs the randomized data. All the ANOVAs were performed through the Python library SciPy (39) and assessed using alpha levels of 0.0001, 0.001, 0.01, and 0.05. Classifiers were also evaluated with receiver operator characteristic (ROC) curves and precision-recall (PR) curves. ROC curves plot the true positive rate against the false-positive rate (FPR). Classifiers with the largest area under the curve (AUC) values have better true-to-false-positive ratios, with AUC values greater than 0.5 indicating better than random ability to correctly predict the presence/absence of a given bioactivity. The PR curves plot the recall (i.e., true positives over the sum of true positives and false negatives) against the precision (i.e., true positives over the sum of the true positives and the false positives); they are considered more accurate for classifiers trained on imbalanced data sets, such as ours.

### Assembling the fungal phylogeny

To analyze the distribution of the characterized SM bioactivities across the fungal kingdom we displayed the different types of bioactivity known for different species on the branch tips of a phylogeny using the Interactive Tree of Life (iToL) tool, version 6 (40). The phylogeny was modified with no tip labels and no branch lengths from a previous phylogenomic analysis of 290 genes from 1,644 fungal species (41). If a species in the data set was not present in the phylogeny, we chose a close relative in the same genus; if the genus was also absent, the data for that species were not displayed on the phylogeny.

## RESULTS AND DISCUSSION

### Classifiers trained on fungal data have low balanced accuracies

**Fig 2:**
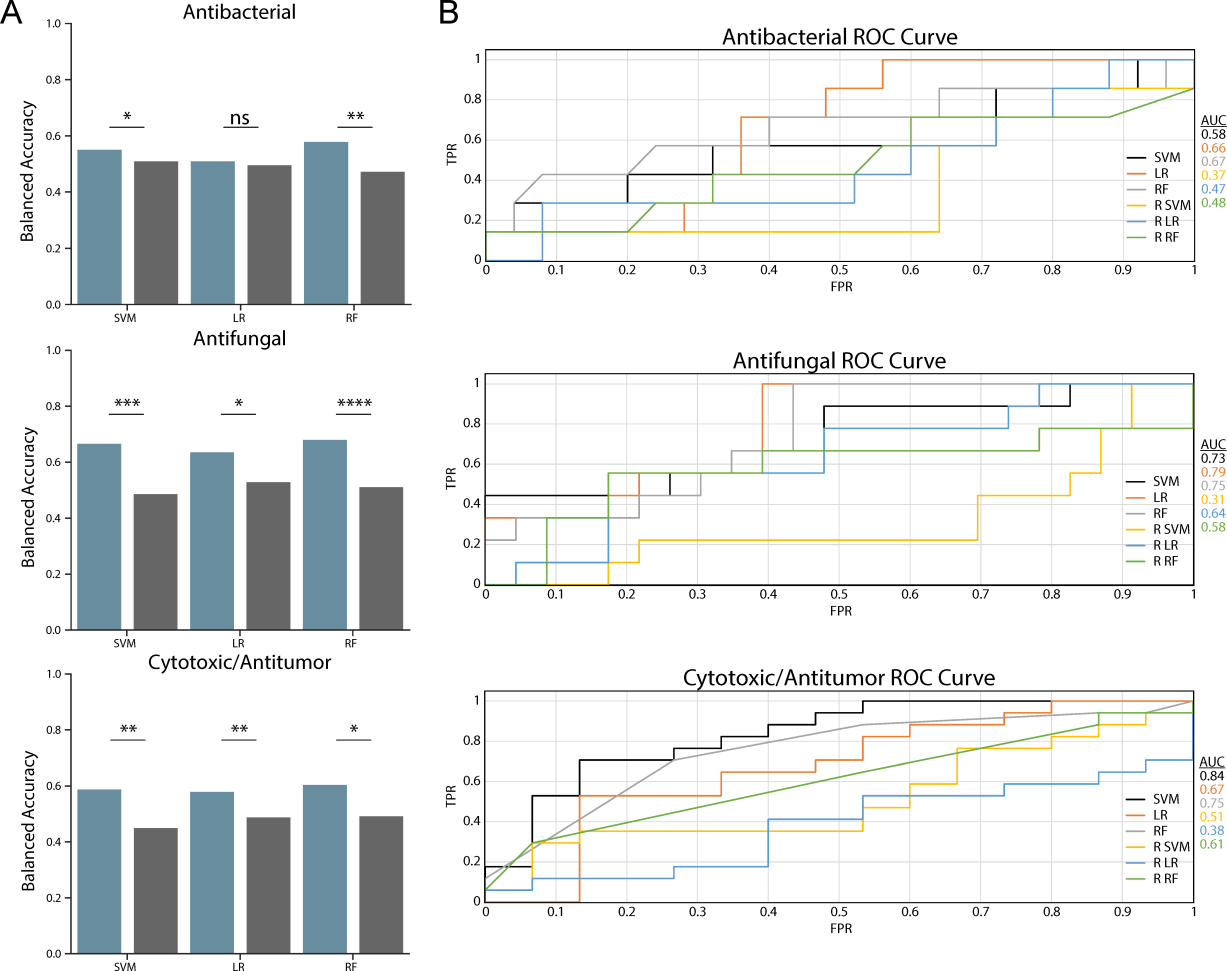
Machine learning models trained on fungal BGC data exhibited low balanced accuracies. (**A**) Balanced accuracy of classifiers. The *x*-axis shows SVM, LR, and RF classifiers trained on actual data (blue) and classifiers trained on randomized features (gray). The *y*-axis shows the balanced accuracy of classifiers. The stars indicate the significance of one-way ANOVA at 0.05(*), 0.01(**), 0.001(***), and 0.0001(****). (**B**) The ROC curves for all classifiers. The *x*-axis shows the FPR, and the *y*-axis shows the TPR. The lines of different colors correspond to the three classifiers trained on actual data (SVM, LR, and RF) and to the three classifiers trained on randomized data (R SVM, R LR, R RF). The AUC is shown to the right for each classifier.

To predict fungal SM bioactivity from BGC data, we trained three machine learning classifiers on 245 features from 314 fungal BGCs. The distribution of bioactivities in the training data was 39% antibacterial, 37% antifungal, and 56% cytotoxic/antitumor. The ANOVA analyses comparing the balanced accuracies of the classifiers trained on training data vs randomized features were significant in all cases apart from the LR for antibacterial predictions (Fig. 2A). The balanced accuracies for antifungal (SVM, 66%; LR, 64%; RF, 68%) were the highest, followed by cytotoxic/antitumor (SVM, 59%; LR, 58%; RF, 61%) and antibacterial (SVM, 55%; LR, 51%; RF, 58%) classifications (Fig. 2A).

To identify potential explanations for the low balanced accuracies observed in our classifications, we examined the PR (better at evaluating data sets with moderate to large class imbalances) and ROC curves (better at evaluating data sets where classes are equal and balanced). Additionally, we analyzed the true positive rates (TPR), false positive rates (FPR), true negative rates (TNR), and false negative rates (FNR) in each of our classifications. We found that the AUCs for the PR curves were consistently lower than the ROC curves for the antibacterial and antifungal classifications (Fig. 2B) (see Fig. S1 at https://doi.org/10.6084/m9.figshare.24129012). This indicated a low number of false positives (explaining why the ROC curves, which plot the FPR against the TPR, but do not account for false negatives, were higher) and a larger number of false negatives (explaining why the PR curves, which plot recall or the true positives/true positives + false negatives against the precision or the true positives/true positives + false positives, were lower). The larger number of false negatives in the antibacterial and antifungal classifications, which is also shown in the confusion matrices from the cross-validation and the average FPR and FNR values (see Fig. S5 to S7; Table S3 at https://doi.org/10.6084/m9.figshare.24129012), suggests that the classifiers are training on the negative class (i.e., on the 0’s) instead of the positive class (1’s) (see Fig. S1 at https://doi.org/10.6084/m9.figshare.24129012). Even though the class of SMs with antibacterial bioactivity is larger than the antifungal class in the training data, the antibacterial classifiers had lower balanced accuracies and higher FNRs compared to the antifungal classifiers. This result could be due to a larger number of shared features between BGCs in the antifungal bioactivity class that increased its prediction accuracy compared to the antibacterial bioactivity class (see Fig. S8 at https://doi.org/10.6084/m9.figshare.24129012). The cytotoxic/antitumor classification had relatively similar ROC and PR AUCs and had high FPRs and TPRs and low FNRs and TNRs (see Table S3 at https://doi.org/10.6084/m9.figshare.24129012). This result suggests that the classifiers are training on the positive class (1’s) but overclassifying 0’s as 1’s, potentially due to the larger number of 1’s for the cytotoxic/antitumor bioactivity in the fungal data set (177 SMs with cytotoxic/antitumor bioactivity out of 314 total) (see Fig. S7 at https://doi.org/10.6084/m9.figshare.24129012). In summary, the relatively low accuracies for all three classifications are likely due to our small, imbalanced training data set.

### Classifiers trained on fungal and bacterial data have similar balanced accuracies

The classifiers trained on fungal data displayed relatively low accuracies compared to classifiers trained on bacterial data from a previous study (30). Thus, we next trained classifiers on a combined data set comprised of both fungal and bacterial data. If the bacterial and fungal BGCs have shared features that correlate with the bioactivity of their SMs in the same way, the combined data set should have increased balanced accuracies in predicting fungal SM bioactivities. However, if there are not enough shared features or they do not correlate to bioactivity, training on the combined data set will result in a decrease in balanced accuracies.

**Fig 3:**
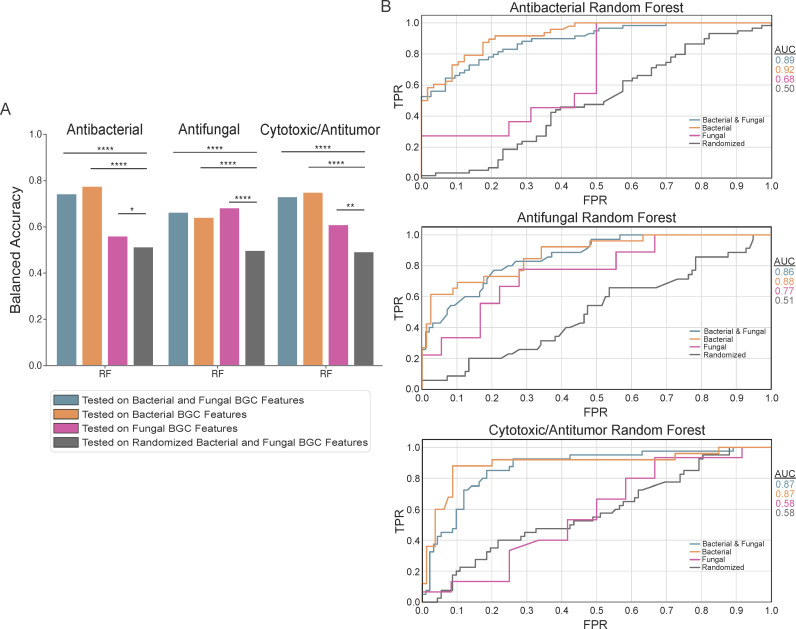
Classifiers trained on both fungal and bacterial BGC data exhibit higher balanced accuracies. (**A**) The *y*-axis shows the balanced accuracies of RF classifiers trained on both fungal and bacterial data. The *x*-axis shows the RF classifier trained on bacterial and fungal data and tested on bacterial and fungal data (blue bars), bacterial data (orange), and fungal data (pink). The gray bars show the RF classifier trained on randomized data and tested on bacterial and fungal data. The stars indicate the significance of one-way ANOVA at 0.05(*), 0.01(**), 0.001(***), and 0.0001(****) for each classifier compared to the classifier trained on randomized features. (**B**)The ROC curves for all RF classifiers. The *x*-axis shows the FPR, and the *y*-axis shows the TPR. The lines of different colors correspond to classifiers trained on both fungal and bacterial data and tested on both fungal and bacterial data (blue), bacterial data (orange), and fungal data (pink); the gray lines correspond to the classifier trained on randomized data and tested on bacterial and fungal data. The ROC curves were selected based on one trial in the 10-fold cross-validation. The AUC values are shown to the right.

There were 984 features in the 1,317 BGCs (1,003 bacterial and 314 fungal). The distribution of bioactivities in the training data was antibacterials, 47%; antifungals, 24%; and cytotoxic/antitumor, 35%. The ANOVA analyses comparing the balanced accuracies of classifiers trained on the training data vs randomized data were all significant (*P* < 0.05) (Fig. 3A) (see Fig. S2 at https://doi.org/10.6084/m9.figshare.24129012). The balanced accuracies of the classifiers trained on both bacterial and fungal data and tested on bacterial and fungal data were between ~61% and ~74%, with the antibacterial classification having the highest balanced accuracies (SVM, 74%; LR, 72%; RF, 74%) followed by cytotoxic/antitumor (SVM, 70%; LR, 68%; RF, 73%) and antifungal (SVM, 64%; LR, 61%; RF, 66%) classifications.

Analysis of this combined data set, where classifiers were trained on both fungal and bacterial data and predicted bioactivity of both bacterial and fungal SMs, revealed that the balanced accuracies were slightly lower compared to the original study by Walker and Clardy (30), where the models were trained on just the bacterial BGC data. The observed differences are potentially due to the differing types of features, such as Pfam domains and cluster-defining features, between fungal and bacterial BGCs, and the imbalanced distributions of bioactivity types in the training data. Walker and Clardy also used sequence similarity networks, which added an additional 825 features to the classifiers trained on bacterial data in their study. However, the removal of these features did not significantly impact accuracies. Compared to the balanced accuracies based on only fungal BGC data, the balanced accuracies based on bacterial and fungal data increased, suggesting that the features in the bacterial and fungal BGCs are similar enough to increase prediction accuracy.

We examined the ROC and PR curves of the models trained on the combined data set and tested on bacterial and fungal data to identify reasons for the reduced accuracy relative to Walker and Clardy. The ROC curves for all classifications had high AUCs with the antibacterial classification (SVM, 0.86; LR, 0.86; RF, 0.89) being the highest, followed by the antifungal (SVM, 0.80; LR, 0.83; RF, 0.86) and cytotoxic/antitumor (SVM, 0.80; LR, 0.80; RF, 0.87) classifications (Fig. 3B) (see Fig. S3 at https://doi.org/10.6084/m9.figshare.24129012). The antibacterial classification had ROC AUC (SVM, 0.86; LR, 0.86; RF, 0.89) values that were similar to the PR AUC (SVM,0.85; LR, 0.85; RF, 0.89) values, and the antifungal classification had ROC AUC (SVM, 0.80; LR, 0.83; RF, 0.86) values that were higher than the PR AUC (SVM, 0.64; LR, 0.66; RF, 0.73) values (see Fig. S4 at https://doi.org/10.6084/m9.figshare.24129012). In contrast, the cytotoxic/antitumor classification had ROC AUCs (SVM, 0.80; LR, 0.80; RF, 0.87) that were substantially higher than PR AUCs (SVM, 0.56; LR, 0.56; RF, 0.72) (see Fig. S4 at https://doi.org/10.6084/m9.figshare.24129012). In general, lower PR AUC values indicate poor performance on the positive class. To explain the lower PR AUC values compared to the ROC AUC values in the cytotoxic/antitumor classification, we analyzed the FPR, TPR, FNR, and TNR. The average FNR for the cytotoxic/antitumor classification was relatively high compared to the FPR (see Table S4 at https://doi.org/10.6084/m9.figshare.24129012) and is likely the reason for the reduction in the PR AUC values as seen in the cross-validation confusion matrices (see Fig. S11 at https://doi.org/10.6084/m9.figshare.24129012). The high FNR in the cytotoxic/antitumor classification could be due to the lack of shared features between fungal and bacterial BGCs.

To determine if the relatively high balanced accuracies observed from the classifiers trained on the bacterial and fungal data sets were driven by the large portion of bacterial BGCs in the training data, we next analyzed the performance of the classifiers trained on the combined data set but tested separately on their ability to predict bacterial SM bioactivity and fungal SM bioactivity in the 10-fold cross-validation. In the antibacterial classification, the classifiers had higher balanced accuracies and better performance on predicting bacterial SM bioactivity (SVM, 78%; LR, 77%; RF, 78%) than fungal SM bioactivity (SVM, 61%; LR, 58%; RF, 56%) (Fig. 3A) (see Fig. S2 at https://doi.org/10.6084/m9.figshare.24129012). This is likely due to their larger number in the combined data set used for training. Interestingly, in the antifungal classification, the balanced accuracies were similar when predicting bacterial (SVM, 63%; LR, 60%; RF, 64%) and fungal SM bioactivity (SVM, 61%; LR, 62%; RF, 68%) (Fig. 3A) (see Fig. S2 at https://doi.org/10.6084/m9.figshare.24129012). Lastly, the classifiers in the cytotoxic/antitumor classification generally had better performance on bacterial SM bioactivity (SVM, 70%; LR, 68%; RF, 75%) than on fungal SM bioactivity (SVM, 64%; LR, 60%; RF, 61%) (Fig. 3A) (see Fig. S2 at https://doi.org/10.6084/m9.figshare.24129012). Additionally, the ROC AUCs were consistently lower for classifiers tested on fungal data compared to classifiers tested on bacterial data (Fig. 3B) (see Fig. S3 at https://doi.org/10.6084/m9.figshare.24129012). This is likely due to the small portion of fungal data in the training data set and the smaller portion of fungal data in the cross-validation (tested on ~30–40 fungal BGCs and ~90–100 bacterial BGCs). The difference in performance between the fungal and bacterial data for each classifier and bioactivity shows that despite the differences in features between fungal and bacterial BGCs, there are sufficient similarities to result in balanced accuracies of fungal bioactivity that do not decrease (and in the case of predicting antifungal bioactivity even slightly increase). Thus, using models trained on both bacterial and fungal data does not reduce the accuracy of predicting fungal SM bioactivity.

### Low balanced accuracies stem from the lack of fungal BGC data

An additional explanation for the lower accuracies of our models trained on fungal and bacterial data relative to the accuracies observed by Walker and Clardy with training on only bacterial data could be that there is substantial diversity in features that contribute to each bioactivity between bacterial and fungal BGCs. For example, there are numerous antibacterial agents that do not share the same targets; some have bacteriostatic or bactericidal properties but have the same mode of inhibition in preventing protein synthesis (42), while others inhibit peptidoglycan biosynthesis to form pores in bacterial membranes. Additionally, some have broad-spectrum or narrow bioactivity against different microbes increasing their specificity. The small size of our data set, especially of fungal BGCs, likely does not sufficiently capture the ways in which different BGC features can affect bioactivity for different mechanisms of action, especially for those that have multiple bioactivity types.

Consistent with this explanation, feature importance analysis showed that the most informative features in the antibacterial and cytotoxic/antitumor bioactivity predictions generally differed between the classifiers trained on fungal data and classifiers trained on fungal and bacterial data (see Fig. S12 and S13 at https://doi.org/10.6084/m9.figshare.24129012). A comparison of the top 25 most informative features of the two data sets showed an overlap of 4 features for the antibacterial classifiers and 8 features for the cytotoxic/antitumor classifiers.

**Fig 4:**
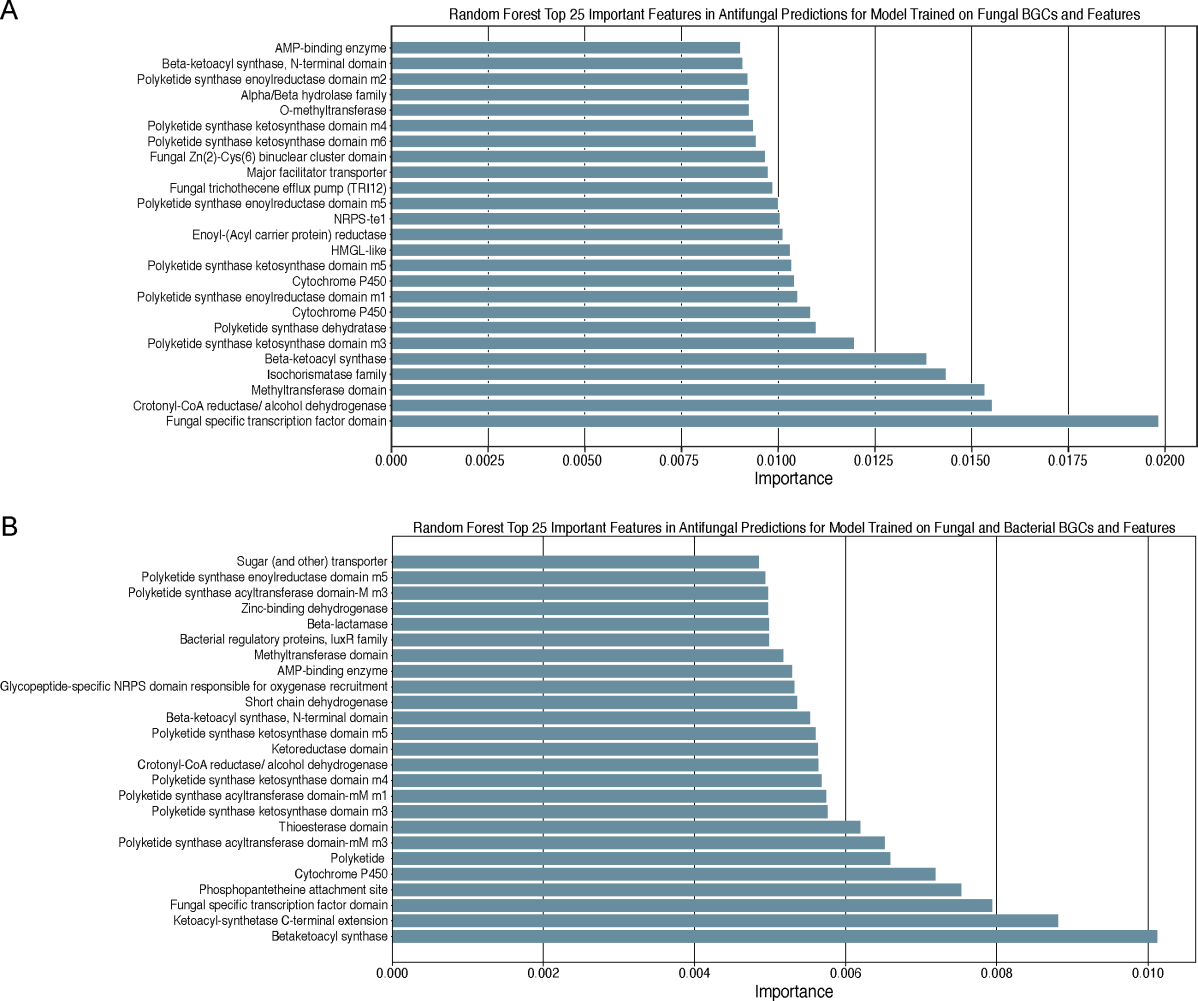
The 25 most important features in antifungal predictions are similar between models trained on just fungal BGC data vs models trained on fungal and bacterial BGC data. (**A**) Top 25 important features in antifungal predictions for the models trained on fungal data. The *x*-axis shows the importance, and the *y*-axis shows the features. (**B**) Top 25 important features in antifungal predictions for the models trained on fungal and bacterial data. The *x*-axis shows the importance, and the *y*-axis shows the features.

In contrast, the most informative features in the antifungal bioactivity predictions had a larger amount of overlap between the two training data sets with 11 of 25 features overlapping. A few of the features that appeared in both data sets were crotonyl-CoA reductase, AMP-binding enzyme, multiple polyketide synthase domains, and fungal-specific transcription factor domain (Fig. 4). This overlap of features between fungal and bacterial BGCs that exhibit antifungal bioactivities may stem from many antifungals targeting the cell membrane or pathways involved in its assembly (e.g., ergosterol biosynthesis) (43). Many of the overlapping features between both data sets are primarily related to their core genes; for example, NRPS and PKS genes are common in both bacterial and fungal data sets as both groups of organisms produce polyketide and non-ribosomal peptide SMs. In the combined data set, there were 35 PKS features; 24 of these were overlapping between fungal and bacterial BGCs, and 11 were unique to the bacterial data set. Additionally, there were 15 NRPS features in the combined data set with 13 overlapping and two unique to the bacterial data set. However, bacterial BGCs may contain multiple NRPSs compared to typically a single NRPS in fungal BGCs; while the domains and specific features of the genes within the BGCs overlap, the architecture of the BGCs and the SM assembly process between bacterial and fungal BGCs differs (11) (see Tables S6 and S7 at https://doi.org/10.6084/m9.figshare.24129012).

Examination of the 25 most informative features reveals the presence of features with identical annotation names (Fig. 4). The features of the identically named annotations are from Pfam (830 annotations) and smCOG (36 annotations). For example, there is a Pfam domain named “Cytochrome P450” and a smCOG group named “cytochrome P450.” While some genes have both annotations, other genes have one but not the other annotation. To test whether these annotations were truly redundant, we removed Pfam or smCOG features from the fungal data set and observed a slight reduction in the balanced accuracies of our classifiers trained on fungal data (see Table S5 at https://doi.org/10.6084/m9.figshare.24129012). These results suggest that, despite being identically named, these features are not redundant or exact duplicates and that the inclusion of both Pfam and smCOG annotations does not negatively impact accuracy.

Nevertheless, there were also features that were specific to the fungal data set. For example, in models trained on only fungal data, the Pfam identifiers HMGL-like and isochorismatase family were present in ~5% of the BGCs with antifungal bioactivity and in 0% of the BGCs without. The HGML-like domain encompasses a family of aldolases and a region of pyruvate carboxylases that all contain phosphate binding sites, and the isochorismatase family is a family of hydrolase enzymes. Both enzymes are involved in conversion steps in metabolic pathways, and isochorismatase family enzymes have been involved in the degradation of creatinine in *Pseudomonas putida* and *Arthrobacter* sp. (44). Additionally, the polyketide synthase ketosynthase domain in module 3 (PKSI-KS m3) of BGCs was present in ~78% of the BGCs with antifungal bioactivity vs ~54% of BGCs without, and the beta-ketoacyl synthase, N-terminal domain, was present in 79% of BGCs with antifungal bioactivity vs ~56% of BGCs without in models trained on fungal data.

### Narrow taxonomic distribution of SM bioactivities across the fungal kingdom

Another potential explanation for the low accuracies of the classifiers trained on fungal BGC data may be that the bioactivities of already characterized fungal SMs stem from only a subset of fungal clades. To analyze the distribution of characterized SM bioactivity types across the fungal kingdom and determine if there are any phylogenetic patterns in the bioactivities we studied, we mapped the bioactivity types to a phylogeny of 1,644 species that span the diversity of the fungal kingdom (41). The 314 fungal BGCs used in our training data stemmed from 78 species that produced BGCs with SM products that had antibacterial bioactivity (53 were in the phylogeny, 5 were not in the phylogeny, and we used another representative from the same genus for 20 species), 81 with antifungal bioactivity (47 in the phylogeny, 7 not in the phylogeny, we used another representative from the same genus for 27 species), 62 with cytotoxic bioactivity (35 in the phylogeny, 5 not in the phylogeny, we used another representative from the genus for 22 species), and 79 with antitumor bioactivity (45 in the phylogeny, 8 not in the phylogeny, we used another representative from the same genus for 24 species).

**Fig 5:**
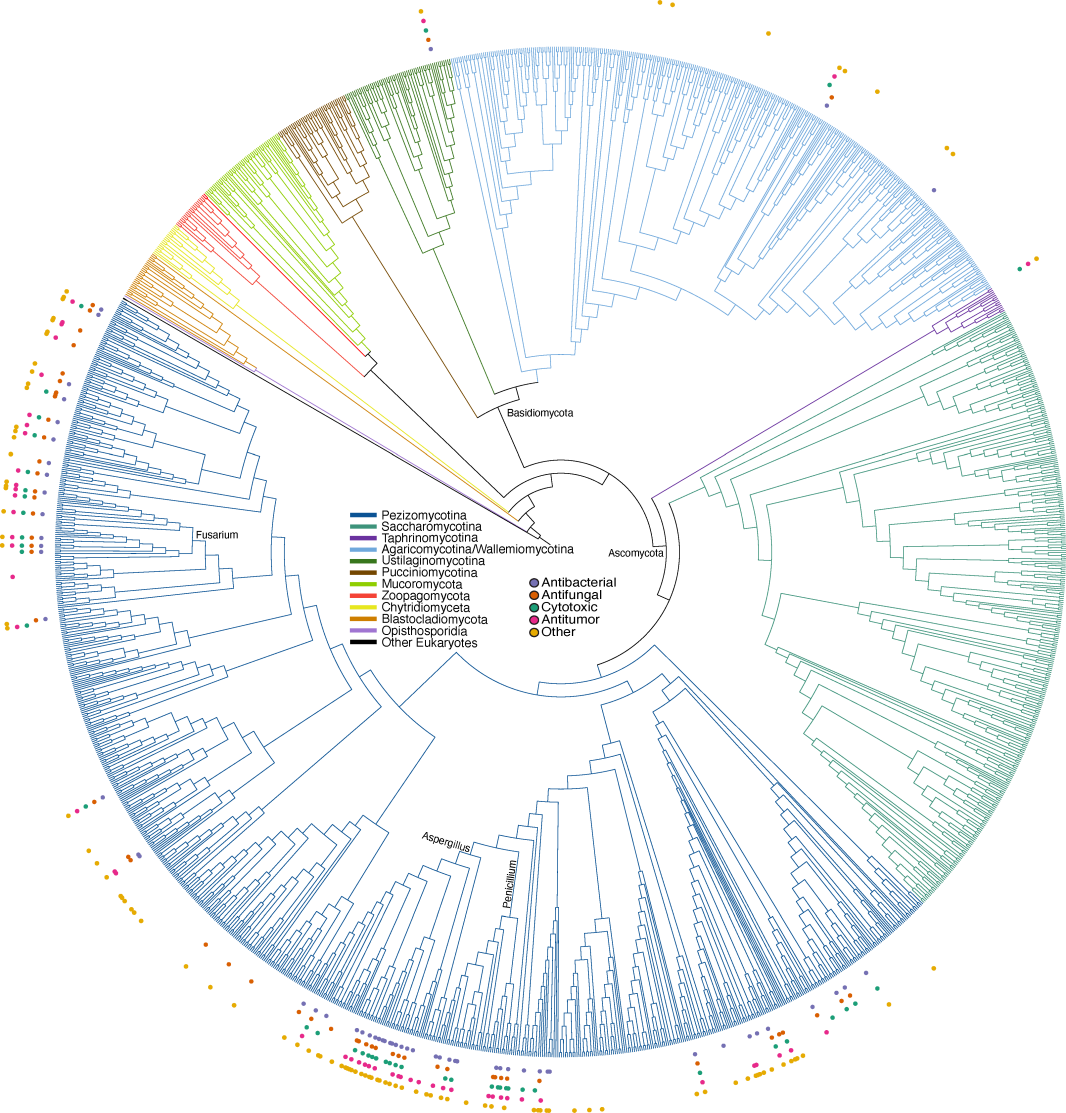
Narrow taxonomic distribution of characterized biosynthetic gene cluster-secondary metabolite pairs across the fungal kingdom. Phylogeny modified from Li et al. (41). The branch colors indicate different subphyla within the fungal kingdom. The circles indicate the four activity types (antibacterial, antifungal, antitumor, and cytotoxic) included in the predictions and other activity types noted in the data set (e.g., antifeedant) as well as the secondary metabolites with unknown bioactivity (others).

While there were not any notable phylogenetic patterns in the distributions of bioactivity types across fungi, there was a very sporadic distribution of characterized BGC-SM pairs with areas of relatively dense coverage (Fig. 5). A few genera were well sampled compared to the rest of the phylogeny, including *Aspergillus*, *Penicillium*, and *Fusarium*; in contrast, BGC-SM pairs with characterized bioactivity were rather sparsely distributed across most of the phylogeny. This was the case even in the *Pezizomycotina* subphylum (phylum Ascomycota) of filamentous fungi, which are well known to contain many BGCs (45).

We analyzed the distribution of genera in the fungal data set and observed that while few genera contained many BGC-SM pairs, there were many that had none or only one representative in the data set (see Fig. S14A at https://doi.org/10.6084/m9.figshare.24129012). Additionally, of the 392 BGC-SM pairs in the original data set and the 314 fungal BGCs used in model training, 131 and 101, respectively, were derived from the genus *Aspergillus*, indicating a large bias in the characterization of fungal BGC-SM pairs. Notably, this taxonomic bias is not unique to fungal BGCs-SM pairs. We observed a very similar distribution in the bacterial data, where over 400 of the bacterial BGCs-SM pairs are from the genus *Streptomyces* (see Fig. S15 at https://doi.org/10.6084/m9.figshare.24129012).

We next used anti-SMASH to determine if the lower number of BGC-SM pairs in certain fungal species or genera was due to smaller numbers of BGCs in their genomes. We found that the number of predicted BGCs across species did not correlate with the number of BGC-SM pairs, suggesting that the lack of representation of certain taxa in the data set is likely due to lack of studies that link these BGCs to their corresponding SMs and their bioactivities (see Fig. S14B at https://doi.org/10.6084/m9.figshare.24129012).

**Fig 6:**
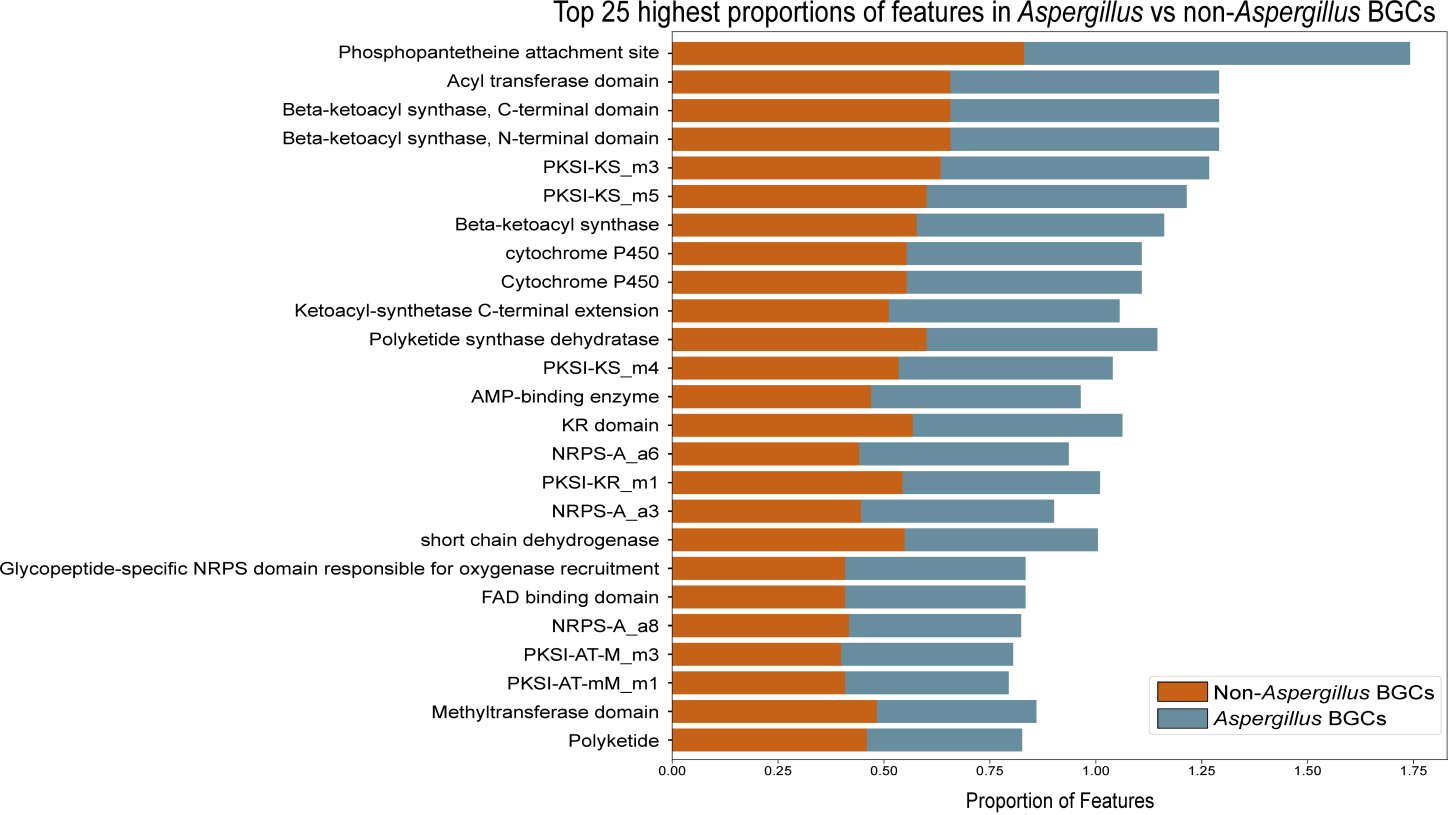
Features in *Aspergillus* BGCs are representative of the diversity of features in the *Pezizomycotina* subphylum. The *x*-axis shows the proportion of feature presence in *Aspergillus* (blue) vs *non-Aspergillus* (orange) BGCs, and the *y*-axis shows all the features in the fungal data set.

Given the bias in characterization clearly depicted in the number of BGC-SM pairs derived from *Aspergillus* and *Streptomyces*, we next examined whether the BGC features from these two genera were representative of the BGC features in the rest of the fungi and bacteria, respectively. Specifically, we compared the proportion of features present in *Aspergillus* and *Streptomyces* BGCs to the proportion of features present in non-*Aspergillus* and non-*Streptomyces*-derived BGCs. In the fungal data set, we observed that the proportions of features between *Aspergillus*-derived BGCs and non-*Aspergillus*-derived BGCs were similar (Fig. 6) (see Fig. S16 at https://doi.org/10.6084/m9.figshare.24129012). This suggests that the features present in *Aspergillus* BGCs are likely representative of the diversity of features in the *Pezizomycotina* subphylum (phylum *Ascomycota*). We observed a similar result in the data set with bacterial and fungal BGCs with notably more features absent in *Streptomyces* BGCs than in the non-*Streptomyces* BGCs (see Fig. S17 at https://doi.org/10.6084/m9.figshare.24129012). These results suggest that the taxonomic bias in BGC-SM pair characterization does not significantly bias the feature types present in the data. Furthermore, in the fungal data specifically, these results suggest that the low accuracy of using machine learning to predict fungal SM bioactivity is due to the small number of characterized fungal BGC-SM pairs rather than the lack of diversity of BGCs from different species in the training data. In other words, both *Aspergillus* and *Streptomyces* genera are potentially good models to use in machine learning due to the current numbers of characterized BGC-SM pairs and the types of features present.

### The need for systematic effort in characterizing BGC-SM pairs

Currently, there have been more than 15,000 fungal SMs characterized and millions of putative BGCs identified in fungal genomes (46). Due to the increased demand for novel drugs, efforts that systematically link fungal SM bioactivity to BGCs are urgently needed. Various methods of correlating SMs to their corresponding BGCs have been undertaken such as targeted genome mining, heterologous expression, metabologenomics with gene cluster family (GCF) networking and correlation-based scoring (47), and feature-based correlation methods utilizing genome-metabolome ontologies in bacterial species (48). While these methods are continuing to improve, SM discovery is still far ahead of the characterization of the BGCs responsible for their biosynthesis. Although there are various databases available for characterized SMs, such as the Dictionary of Natural Products and Medicinal Fungi Secondary metabolites And Therapeutics (MeFSAT) (49), there remain potentially millions of uncharacterized BGC-SM pairs. Publishing the results of SM bioactivity assays in repositories, including the negative results, would be another systematic effort that could enable larger analyses and greater accessibility to these data. Only a small portion of SMs have been linked to BGCs, and an even smaller portion of these BGC-SM pairs have characterized bioactivities. Considering more than 15,000 fungal SMs known to date and new methodologies for linking SMs and BGCs (20), the potential opportunities and benefits of filling this large gap of knowledge are considerable.

Machine learning methods that predict fungal SM bioactivity from BGC data show promise but are currently unable to perform with high accuracy due to the lack of fungal BGC-SM pairs with bioactivity data. Here, we obtained low balanced accuracies for models trained on fungal BGCs (51%–68%), likely because only 314 fungal BGCs were used to train our models. Additionally, while the BGC-SM pairs within MIBiG are experimentally validated, not all genes in each BGC have been experimentally validated. The lack of validation of each BGC is a limitation to this study as the input for model training is BGC data and relies on the genes within the BGCs to make informed predictions on bioactivity. There are BGCs that modify existing compounds, rather than *de novo* assembling the entire SM, increasing the complexity of predicting SM bioactivity from BGC data.

Walker and Clardy (30) showed that using a data set with 1,003 bacterial BGCs achieved accuracies as high as ~80%, so we hypothesize that at least ~1,000 fungal BGC-SM pairs with known bioactivities, will be necessary to substantially increase accuracy. Incorporating additional features related to the chemical structures of the SMs could increase the specificity between bioactivity classes and increase the balanced accuracies. A previous study examined the scaffold diversity of fungal SMs (50), suggesting that complex SM structures could be broken down into different features that capture scaffold content and structural diversity. There are numerous methods of converting chemical structures into machine-readable formats like simplified molecular input line entry system representation (SMILES) and molecular fingerprinting, which could enable their direct application into machine learning and advance the understanding of chemical diversity (51).

### Conclusions

We used machine learning models trained on fungal BGC data, as well as both fungal and bacterial BGC data to predict fungal SM bioactivity. Due to the dearth of data on fungal BGC-SM pairs and the lack of informativeness of bacterial data for predicting fungal SM bioactivity, our models exhibited relatively low balanced accuracies. Further model optimization for predicting fungal SM bioactivity will require a larger data set of fungal BGC-SM pairs. Additionally, breaking down the large, generalized bioactivity types (antibacterial, antifungal, cytotoxic/antitumor) into more specific classes incorporating the target or mode of action in addition to chemical structure features may aid in more specificity and better accuracies in predicting the bioactivity (although even larger numbers of BGC-SM pairs may be required). Ultimately, improving the model’s overall performance and accuracy will require a systemic effort in characterizing BGC-SM pairs and their bioactivities (including negative results) and depositing the data in large, publicly available repositories. While the current accuracies of artificial intelligence approaches are constrained by the lack of sufficient training data, the potential of machine learning applications in fungal secondary metabolism will remain untapped.

## Data Availability

All supplementary tables and figures are available at https://doi.org/10.6084/m9.figshare.24129012. All input files and scripts required to reproduce the results of this study are available at https://doi.org/10.6084/m9.figshare.24129012.
